# Supplementary material for: Magnetic fields and relativistic electrons fill entire galaxy cluster
Source: Sci Adv. 2022 Nov 2;8(44):eabq7623. doi: 10.1126/sciadv.abq7623 (PMC9629711; doi:10.1126/sciadv.abq7623)
Supplement: Supplementary file 1 — Supplementary Text Figs. S1 to S7 Table S1 References [file sciadv.abq7623_sm.pdf]

Science Advances
AAAS

Supplementary Materials for

Magnetic fields and relativistic electrons fill entire galaxy cluster

Andrea Botteon *et al.*

Corresponding author: Andrea Botteon, botteon@strw.leidenuniv.nl

Sci. Adv. **8**, eabq7623 (2022)
DOI: 10.1126/sciadv.abq7623

This PDF file includes:

Supplementary Text
Figs. S1 to S7
Table S1
References

Supplementary materials

Energy of non-thermal components. The observed synchrotron radiation in the cluster outskirts allows us to estimate an energy budget in the form of relativistic components. The energy density of particles and field is:

$$\epsilon = \frac{B^2}{8\pi} + (1+k)\int_{\gamma_{min}} EN(\gamma)d\gamma \,, \tag{S1}$$

where k is the energy ratio of relativistic protons and electrons. We assume a spectrum of relativistic electrons in the form:

$$N(\gamma) = K_e \gamma^{-\delta} \,. \tag{S2}$$

Of course, our observations do not allow us to measure this spectrum, yet we can safely assume that the real spectrum is matched by such a power law distribution at least in a suitable range around the energy of the electrons that are necessary to radiate at the frequencies where emission is detected; in particular, we note that synchrotron radiation at 49 MHz is generated by electrons with kinetic energy $E_{kin} \approx 1.5 B_{\mu G}^{-1/2}$ GeV (that is $\gamma \sim 3000$ for μG field level).

The minimum energy that is necessary to generate the observed radiation is:

$$\epsilon_{min} = \frac{B^2_{min}}{8\pi}(1+\mathcal{R}) \,, \tag{S3}$$

where the ratio of CR and B energy densities is (Eq. 40 in (*56*)):

$$\mathcal{R} = \frac{2}{1+\alpha} \,, \tag{S4}$$

with $\alpha = (\delta - 1)/2$, and

$$B_{min}(\gamma_{min}, k, ..) = \left(C_\alpha \frac{L_{syn}(\nu)}{V} \nu^\alpha (1+k) \right)^{\frac{1}{3+\alpha}} \gamma_{min}^{\frac{1-2\alpha}{3+\alpha}} \,, \tag{S5}$$

where $L_{syn}(\nu)$ is the synchrotron luminosity at frequency ν, V the emitting volume, and C_α a constant (that is 2.44×10^6 for $\alpha = 1.6$, see Appendix A in (*57*) for more details). Assuming a cylinder $1{\times}1$ arcsec$^2{\times}$ 1.5 Mpc, a brightness at 49 MHz $\sim 2\mu$Jy arcsec^{-2} measured at 1.5$-$2 Mpc distance (Fig. S7, panel b), and the observed $\alpha = 1.6$, we obtain $B_{min} \simeq 0.45(1+k)^{0.222}(\gamma_{min}/1000)^{-0.409}\mu$G.

This implies $\epsilon_{min} \sim 2 \times 10^{-14}$ erg cm^{-3}, that should be compared to the thermal energy density measured in the same region $\epsilon_{ICM} \sim 3P_e \sim 4 \times 10^{-13}$ erg cm^{-3} (P_e is provided in Fig. 4 of (*13*)). The magnetic field could be smaller if electrons have a larger energy budget, yet the total energy budget of the relativistic plasma will increase and become significant with respect to the thermal ICM.

Assuming a departure from minimum energy $B = B_{min}\Delta$, and using the condition $K_e B^{(\delta+1)/2} = K_{e,min} B_{min}^{(\delta+1)/2}$ (i.e. fixed synchrotron luminosity), the energy density is:

$$\epsilon = \epsilon_{min} \frac{\Delta^{-2} + \mathcal{R}\Delta^{1+\alpha}}{1+\mathcal{R}} \tag{S6}$$

consequently, adopting $\alpha = 1.6$, $B \sim 0.1$ μG (that is $\Delta \sim 0.2$) would imply that relativistic electrons keep an energy budget similar to that of the ICM, that is an untenable condition; a similar conclusion is obtained for $B \sim 1.7$ μG (that is $\Delta \sim 3.8$) in which case the magnetic field would keep an energy budget similar to the ICM. Thus, the observed synchrotron radiation implies a lower limit of a few 0.1 μG, which is $>$10 times larger than the value of the primordial field compressed at a cosmic density $\sim$100 times the average value of the Universe. We note that our conclusions remain essentially the same, within a factor 2, if we adopt $\alpha \sim 1.4 - 1.7$ and $\gamma_{min} \sim 1000 - 3000$.

The synchrotron radiation detected by LOFAR at 49 MHz and 145 MHz is essentially contributed by electrons with energies of few GeV. An important point to understand is also how far the spectrum of these electrons can be extrapolated at low energies. This point is relevant

to understand the origin of the particles, for example in the case of direct acceleration of the particles from the thermal pool it is necessary that the observed spectrum extends to the tail of the thermal distribution (*58*). The minimum energy of the relativistic plasma is obtained combining Eqs. S3 and S5 and it depends on the γ_{min} of the spectrum of electrons and on the spectrum, $\delta = 2\alpha + 1$. In Fig. S7 we show the observed radio brightness at 49 MHz and 145 MHz and the spectral index α along the NW and S directions extracted from $60''$ images after subtraction of discrete sources (except for the Beaver, Embryo, Goldfish, Original TRG, Trail, and T-bone radio galaxies, see Methods and Materials). Using the values of spectral indices and brightness in Fig. S7 and Eq. S5 (brightness in Fig. S7 is used to calculate the emissivity L_{syn}/V in Eq. S5 assuming a column length of 1.5 Mpc) we find that a minimum kinetic energy of electrons $>$20$-$30 MeV is required at distances 1.2$-$1.8 Mpc (considering the NW profile) to prevent that the minimum energy budget of non-thermal components matches the thermal energy budget. This simply implies that the observed spectrum of radio emitting electrons (with energies of a few GeV) cannot extend at energies $\leq$ several MeV, and thus that the radio emitting electrons are not accelerated from the thermal pool but extracted (re-accelerated) from a reservoir of particles that are already relativistic.

Electron acceleration and magnetic field amplification efficiencies. The observed synchrotron luminosity also allows us to derive constraints on the efficiency of magnetic field amplification and particle acceleration in the turbulent ICM; specifically we are interested on the possibility to obtain constraints at 1.5$-$2 Mpc distance from the cluster center where the steep spectrum envelope is observed.

The total non-thermal (synchrotron and inverse Compton) luminosity is:

$$L_{NT} = f L(\nu_{49})\nu_{49} \left(1 + \frac{B_{cmb}^2}{B^2}\right) \,, \qquad \text{(S7)}$$

where $B_{cmb} = 3.25(1+z)^2 \mu$G is the inverse Compton equivalent field, $L(\nu_{49})$ is the synchrotron luminosity measured at 49 MHz, and $fL(\nu_{49})\nu_{49}$ gives the bolometric synchrotron luminosity, $L_s = \int d\nu L_{syn}(\nu)$. Specifically, assuming a syncrotron spectrum $L_{syn}(\nu) \propto \nu^{-\alpha}$, $f = [(\frac{\nu_{max}}{\nu_{49}})^{1-\alpha} - (\frac{\nu_{min}}{\nu_{49}})^{1-\alpha}]/(1-\alpha)$, that is $f \sim 5$ considering $\alpha \sim 1.6$ and a minimum frequency $\nu_{min} \sim 5 - 10$ MHz (for steep spectra the value of ν_{max} is not important, provided that $\nu_{max} \gg \nu_{min}$).

If electrons are re-accelerated by turbulence in the ICM, the observed radiation is maintained by a fraction, η_{acc}, of the turbulent kinetic energy flux that is transferred to particles and eventually radiated:

$$\eta_{acc} F V = \frac{1}{2} \eta_{acc} \rho \frac{\sigma_v^3}{\Lambda} V \sim V \int dp \frac{\partial N}{\partial t} E \sim L_{NT} \, , \tag{S8}$$

where V is the emitting volume. As a consequence the fraction can be estimated from the observed synchrotron luminosity and from the turbulent energy flux that is measured in numerical simulations:

$$\eta_{acc} \sim \frac{fL(\nu_{49})\nu_{49}}{FV} \left(1 + \frac{B_{cmb}^2}{B^2}\right) \, . \tag{S9}$$

The same turbulence can amplify the magnetic field via turbulent dynamo (*17*), which stretches and folds magnetic field lines as a consequence of turbulent motions. For large Reynolds numbers the amplification proceeds in a non-linear regime where the magnetic field (Reynolds stress) becomes dynamically important with respect to the turbulent motions on scales that are important for the amplification (*20*). In this case the magnetic field energy density grows linearly with time and the energy density that is converted into magnetic field in an eddy turnover time is:

$$\frac{B^2}{8\pi} \sim \eta_B F \tau_{edd} \, , \tag{S10}$$

where $\tau_{edd} \sim \Lambda/\sigma_v$ is the eddy turnover time and η_B is the fraction of turbulent energy flux that is converted into field in an eddy turnover-time; $\eta_B \sim 0.03 - 0.05$ is measured in MHD simulations (*18, 20, 59*). Combining Eqs. S9 and S10 gives:

$$\eta_{acc} \sim \frac{fL(\nu_{49})\nu_{49}}{FV}\left[1 + \frac{B_{cmb}^2}{8\pi F(\Lambda/\sigma_v)\eta_B}\right] \qquad \text{(S11)}$$

that is used to obtain Fig. S5 with the following parameters: flux density of the envelope 5 Jy, $f = 5$, $V = 10$ Mpc^3, $\Lambda = 200$ kpc, $\sigma_v = 250$ km s^{-1} (from our simulations used in Figs. 1 and S4) and using the solenoidal turbulent energy flux $F \sim 7 \times 10^{43}$ erg s^{-1} Mpc^{-3} from Fig. S4 (we neglect the compressive part because it is a subdominant component). Specifically in Fig. S5 we also show the uncertainties in η_{acc} deriving from a range $FV \sim 3 - 15 \times 10^{44}$ erg s^{-1} (and the corresponding range in σ_v). Flux density and volume of the envelope are estimated considering the region shown in Fig. 2 (removing the contribution of the halo, relic, and discrete sources); the uncertainties on these values are well within the confidence regions reported in Fig. S5.

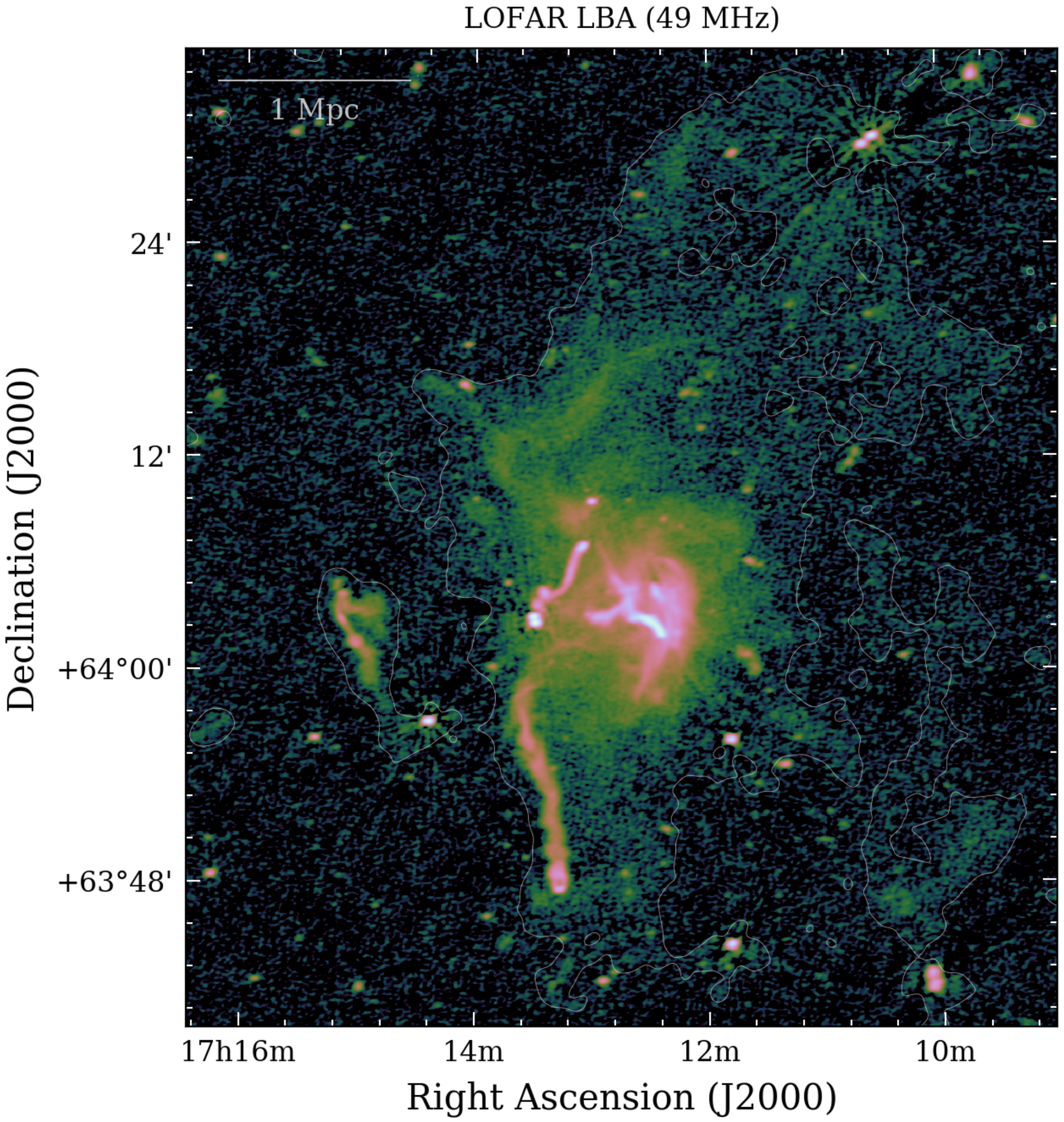

Figure S1: **LOFAR 49 MHz radio image.** The image has a rms noise of 0.48 mJy/beam and a resolution of $20.0'' \times 12.2''$. Colors represent intensity of radio emission. The white contours denotes the 3σ level (with $\sigma = 1.50$ mJy/beam) from the LOFAR 49 MHz image at $60''$ resolution after subtraction of discrete sources (except for the Beaver, Embryo, Goldfish, Original TRG, Trail, and T-bone radio galaxies, see Methods and Materials).

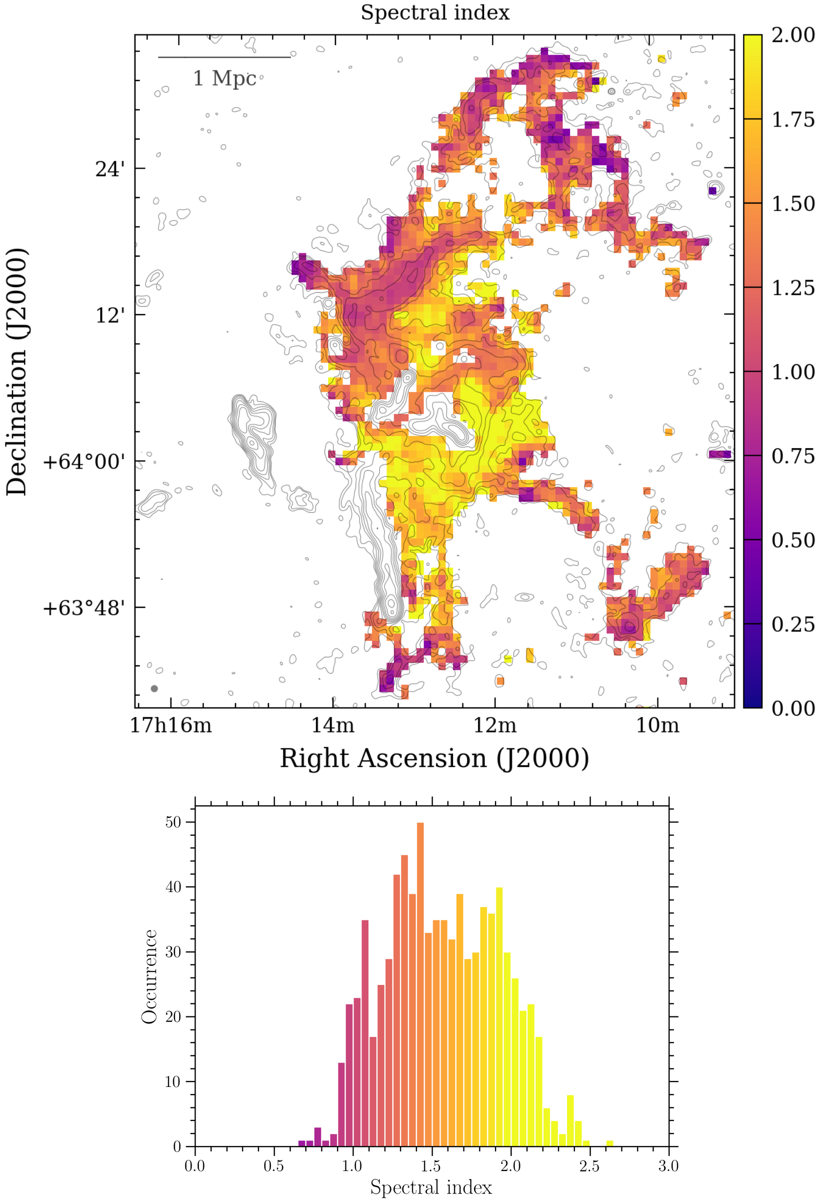

Figure S2: **Spectral index map and spectral index distribution in the range 49-145 MHz.** The map is obtained using LOFAR images at a common resolution of $35''$ (corresponding to 53 kpc at the redshift of Abell 2255) with discrete sources subtracted. Regions with surface brightness values below 2σ in either the two images and/or associated to the bright tailed radio galaxies that were not subtracted in the *uv*-plane were blanked. Black contours represent the emission at 145 MHz, and are spaced by a factor of 2 from 2σ, where $\sigma = 200$ μJy/beam. The beam is shown in the bottom left corner. The histogram refers to regions where $\Delta\alpha/\alpha < 0.2$. The color scale of the spectral index map and histogram is matched.

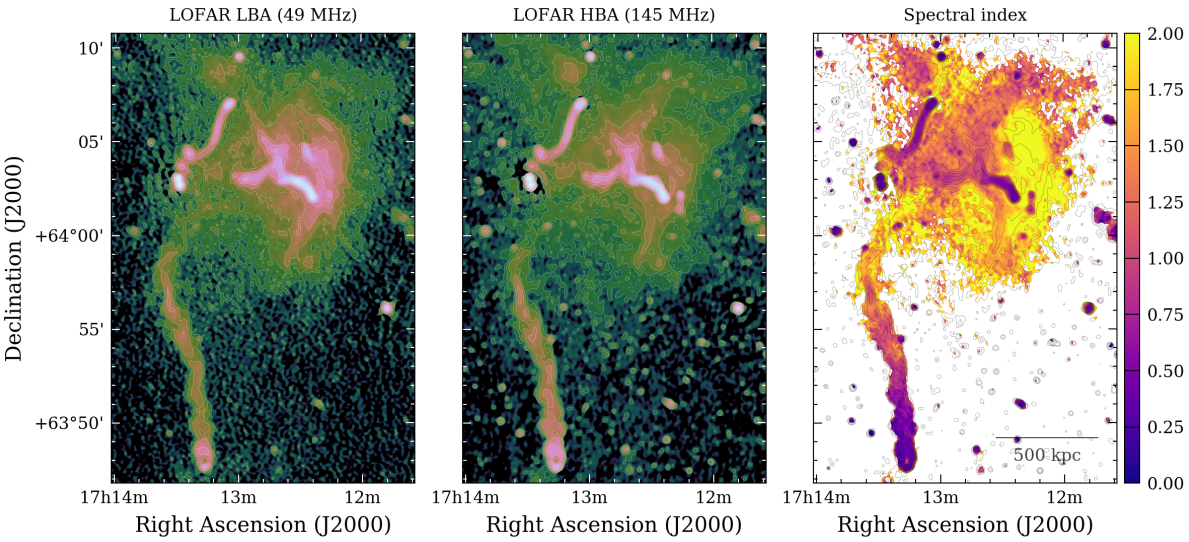

Figure S3: **Images and spectral index map in the range 49-145 MHz.** The images have a common resolution of $12.5''$ (corresponding to 19 kpc at the redshift of Abell 2255). Pixels with surface brightness values below 3σ in the two images were blanked in the spectral index maps. Black contours represent the emission at 145 MHz, and are spaced by a factor of 2 from 3σ, where $\sigma = 80$ μJy/beam. The beam is shown in the bottom left corner.

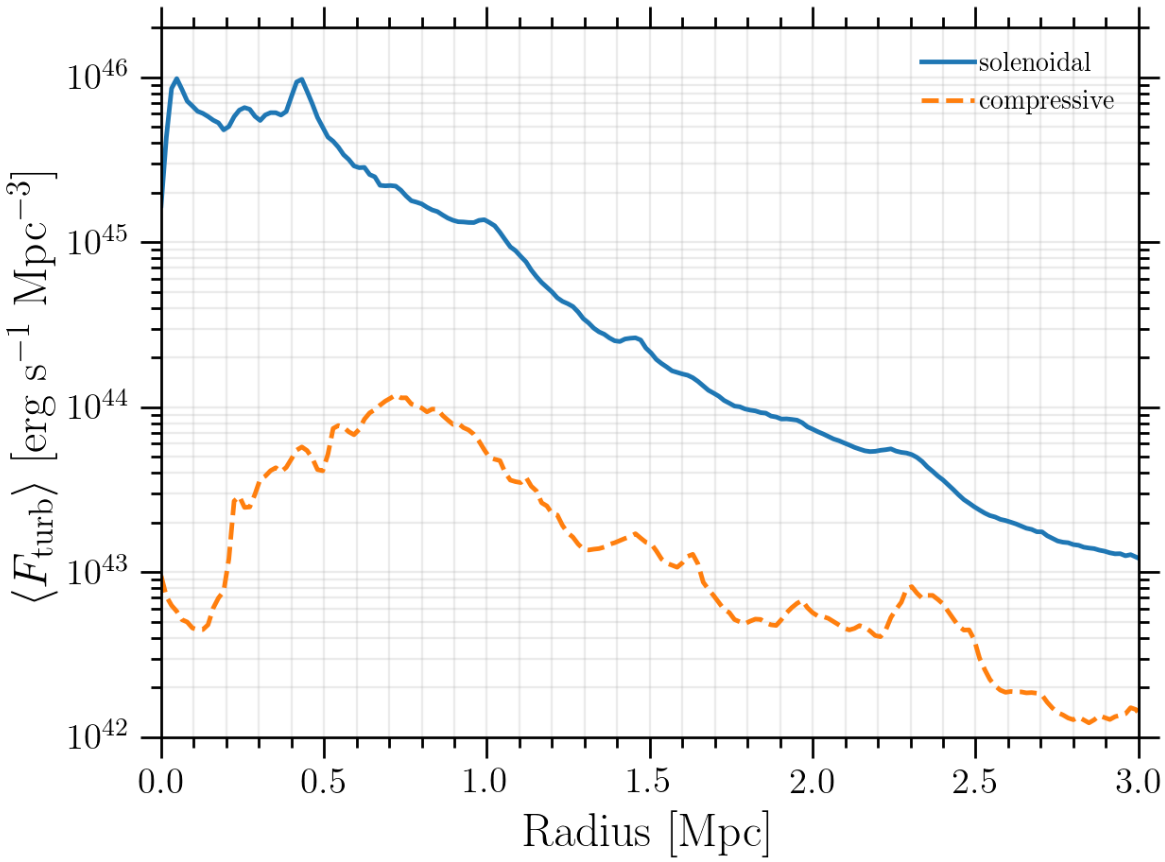

Figure S4: **Radial profiles of the median turbulent energy flux in simulations.** This was obtained from the simulated galaxy cluster of Fig. 1. The two lines show the solenoidal (solid) and compressive (dashed) components of the turbulent energy flux.

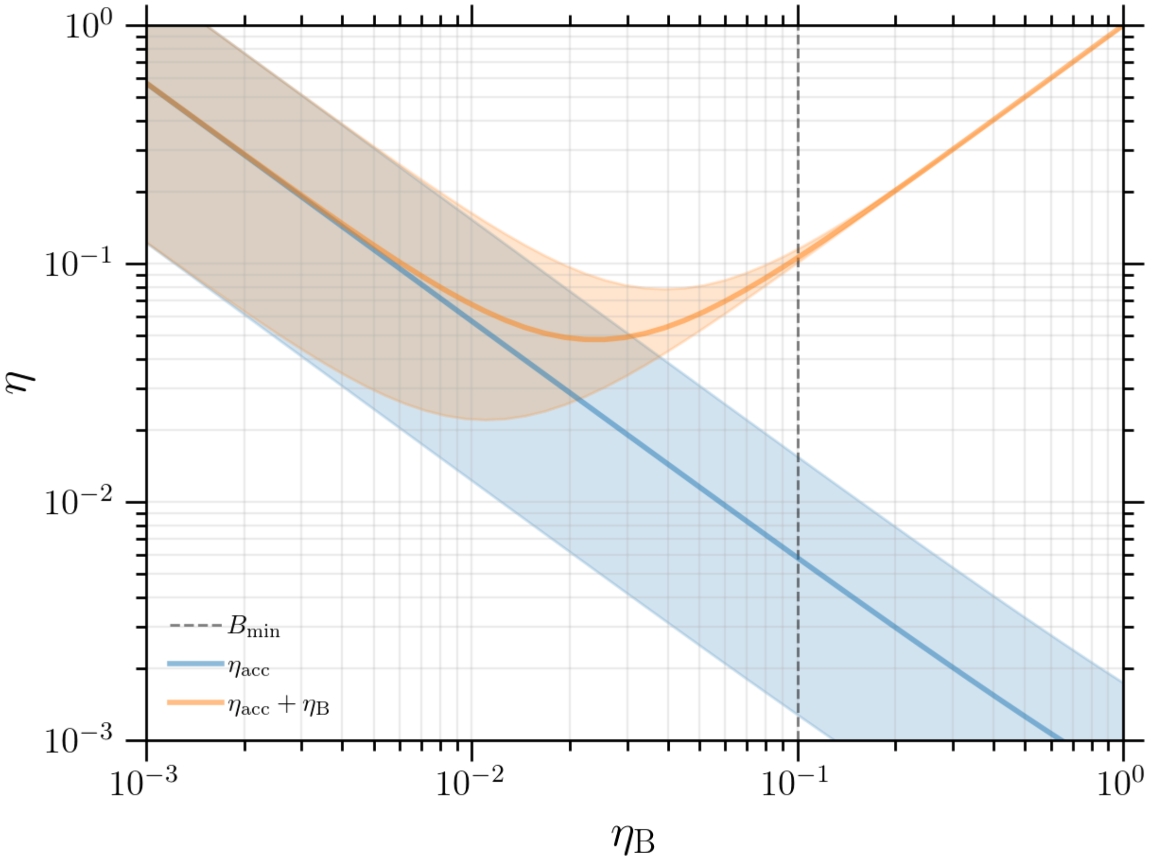

Figure S5: **Efficiency plot.** The solid lines show the fraction of turbulent energy that is converted into particle acceleration η_{acc} (or into acceleration and magnetic field) with corresponding uncertainties (shaded regions) as a function of that converted into dynamo amplification of the magnetic field η_B. The dashed vertical line indicates the magnetic field $B_{min} = 0.45\ \mu$G.

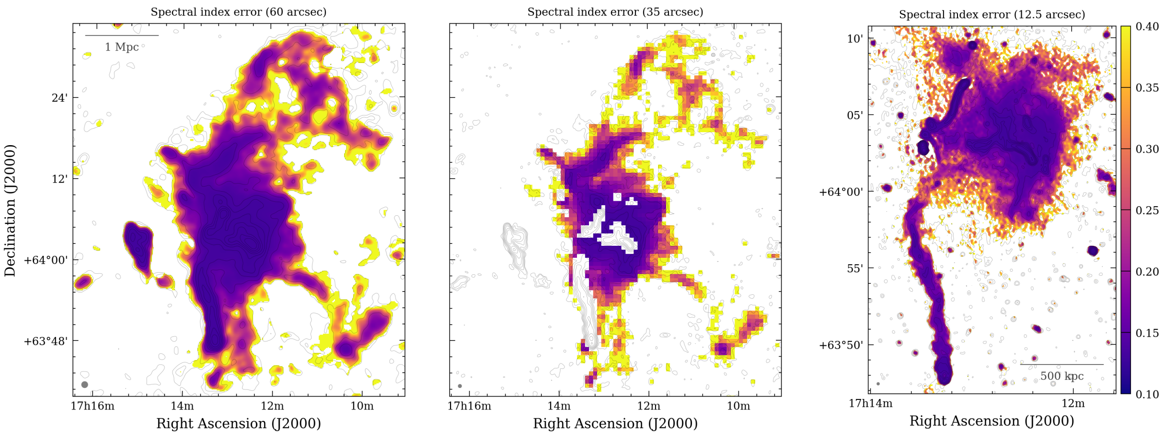

Figure S6: **Spectral index error maps in the range 49-145 MHz.** The images refer to the spectral index maps reported in Figs. 5 (left), S2 (center), and S3 (right). The color scale of the error maps is matched.

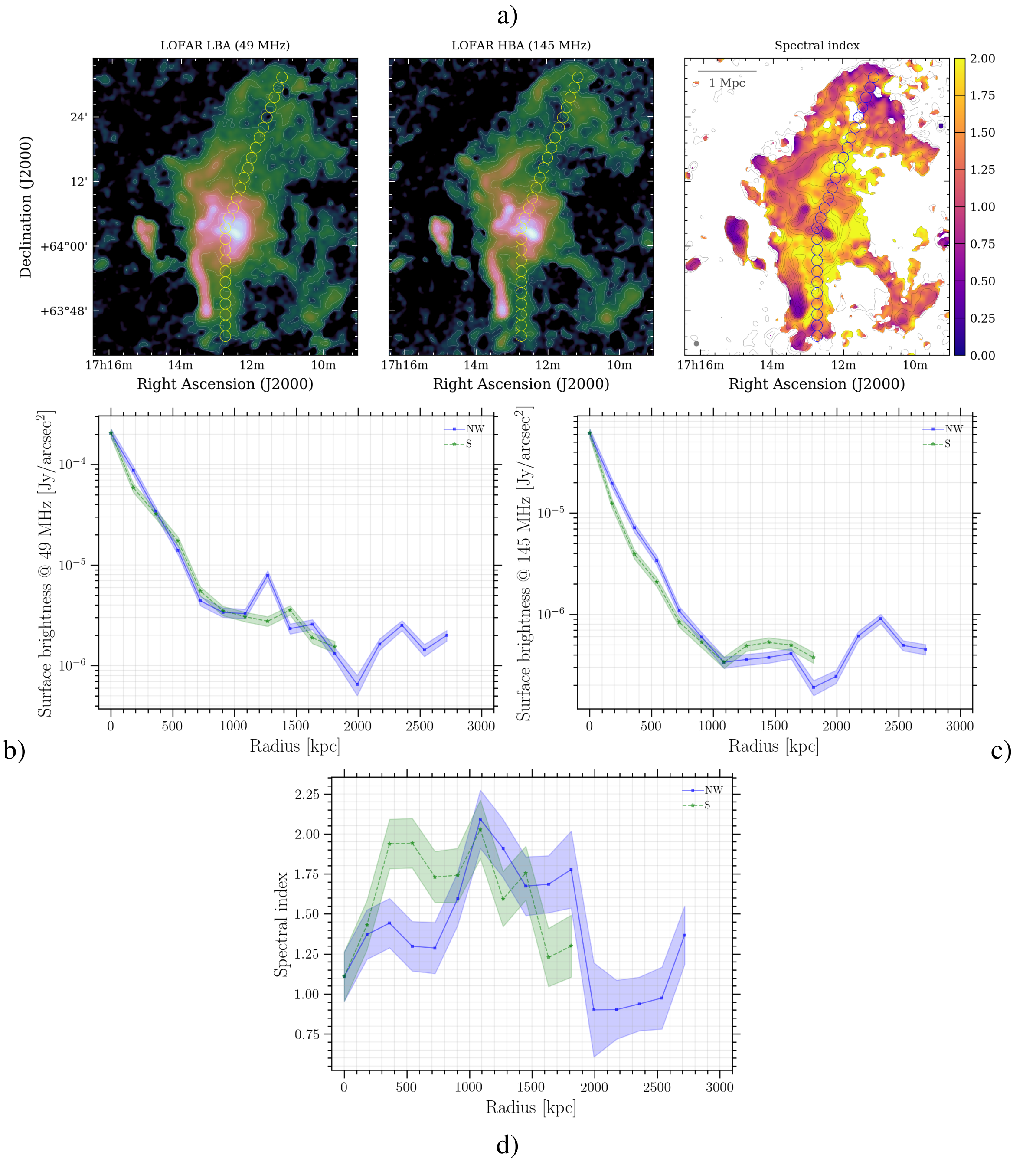

Figure S7: **Radial profiles of the surface brightness and spectral index in Abell 2255.** (a) Regions along the NW and S directions used to extract the profiles overlaid onto the 60″ resolution images at 49 and 145 MHz after subtraction of discrete sources (except for the Beaver, Embryo, Goldfish, Original TRG, Trail, and T-bone radio galaxies, see Methods and Materials) and spectral index map of Fig. 5. (b) Surface brightness profiles at 49 MHz. (c) Surface brightness profiles at 145 MHz. (d) Spectral index profiles in the range 49-145 MHz.

LBA		HBA		Notes
Resolution ($'' \times ''$)	Noise (mJy/beam)	Resolution ($'' \times ''$)	Noise (μJy/beam)	
11.5×8.2	0.73	4.7×3.5	55	Obtained with robust=-1.25
12.5×12.5	0.57	12.5×12.5	80	Obtained with robust=-1.25
20.0×12.2	0.48	7.6×4.7	43	–
35.0×35.0	0.80	35.0×35.0	200	Discrete sources subtracted
60.0×60.0	1.50	60.0×60.0	300	Discrete sources subtracted

Table S1: Resolution and noise of the 49 MHz (LBA) and 145 MHz (HBA) images produced in the paper.

REFERENCES AND NOTES

1. R. J. van Weeren, F. de Gasperin, H. Akamatsu, M. Brüggen, L. Feretti, H. Kang, A. Stroe, F. Zandanel, Diffuse radio emission from galaxy clusters. *Space Sci. Rev.* **215**, 16 (2019).

2. G. Brunetti, T. W. Jones, Cosmic rays in galaxy clusters and their nonthermal emission. *Int. J. Mod. Phys.* **23**, 1430007 (2014).

3. F. Vazza, T. W. Jones, M. Brüggen, G. Brunetti, C. Gheller, D. Porter, D. Ryu, Turbulence and vorticity in Galaxy clusters generated by structure formation. *MNRAS* **464**, 210–230 (2017).

4. R. F. Pizzo, A. G. de Bruyn, Radio spectral study of the cluster of galaxies Abell 2255. *A&A* **507**, 639–659 (2009).

5. D. E. Harris, V. Kapahi, R. D. Ekers, Westerbork synthesis observations of 8 clusters of galaxies which contain tailed radio galaxies. *A&AS* **39**, 215–233 (1980).

6. L. Feretti, H. Böhringer, G. Giovannini, D. Neumann, The radio and x-ray properties of Abell 2255. *A&A* **317**, 432–440 (1997).

7. R. F. Pizzo, A. de Bruyn, L. Feretti, F. Govoni, Detection of diffuse radio emission at large distance from the center of the galaxy cluster A 2255. *A&A* **481**, L91–L94 (2008).

8. A. Botteon, G. Brunetti, R. J. van Weeren, T. W. Shimwell, R. F. Pizzo, R. Cassano, M. Iacobelli, F. Gastaldello, L. Bîrzan, A. Bonafede, M. Brüggen, V. Cuciti, D. Dallacasa, F. de Gasperin, G. Di Gennaro, A. Drabent, M. J. Hardcastle, M. Hoeft, S. Mandal, H. J. Röttgering, A. Simionescu, The beautiful mess in Abell 2255. *ApJ* **897**, 93 (2020).

9. R. Blandford, D. Eichler, Particle acceleration at astrophysical shocks: A theory of cosmic ray origin. *Phys. Rep.* **154**, 1–75 (1987).

10. F. Govoni, M. Murgia, L. Feretti, G. Giovannini, D. Dallacasa, G. B. Taylor, A2255: The first detection of filamentary polarized emission in a radio halo. *A&A* **430**, L5–L8 (2005).

11. R. F. Pizzo, A. de Bruyn, G. Bernardi, M. A. Brentjens, Deep multi-frequency rotation measure tomography of the galaxy cluster A2255. *A&A* **525**, A104 (2011).

12. H. Akamatsu, M. Mizuno, N. Ota, Y.-Y. Zhang, R. J. van Weeren, H. Kawahara, Y. Fukazawa, J. S. Kaastra, M. Kawaharada, K. Nakazawa, T. Ohashi, H. J. Röttgering, M. Takizawa, J. Vink, F. Zandanel, *Suzaku* observations of the merging galaxy cluster Abell 2255: The northeast radio relic. *A&A* **600**, A100 (2017).

13. V. Ghirardini, D. Eckert, S. Ettori, E. Pointecouteau, S. Molendi, M. Gaspari, M. Rossetti, S. De Grandi, M. Roncarelli, H. Bourdin, P. Mazzotta, E. Rasia, F. Vazza, Universal thermodynamic properties of the intracluster medium over two decades in radius in the X-COP sample. *A&A* **621**, A41 (2019).

14. R. Durrer, A. Neronov, Cosmological magnetic fields: Their generation, evolution and observation. *Astron. Astrophys. Rev.* **21**, 62 (2013).

15. K. Subramanian, The origin, evolution and signatures of primordial magnetic fields. *Rep. Prog. Phys.* **79**, 076901 (2016).

16. D. Paoletti, J. Chluba, F. Finelli, J. A. Rubiño-Martín, Improved CMB anisotropy constraints on primordial magnetic fields from the post-recombination ionization history. *MNRAS* **484**, 185–195 (2019).

17. A. Beresnyak, F. Miniati, Turbulent amplification and structure of the intracluster magnetic field. *ApJ* **817**, 127 (2016).

18. F. Vazza, G. Brunetti, M. Brüggen, A. Bonafede, Resolved magnetic dynamo action in the simulated intracluster medium. *MNRAS* **474**, 1672–1687 (2018).

19. P. Domínguez-Fernández, F. Vazza, M. Brüggen, G. Brunetti, Dynamical evolution of magnetic fields in the intracluster medium. *MNRAS* **486**, 623–638 (2019).

20. A. Beresnyak, Universal nonlinear small-scale dynamo. *Phys. Rev. Lett.* **108**, 35002 (2012).

21. J. Cho, Origin of magnetic field in the intracluster medium: Primordial or astrophysical? *ApJ* **797**, 133 (2014).

22. S. Xu, A. Lazarian, Nonlinear turbulent dynamo during gravitational collapse. *ApJ* **899**, 115 (2020).

23. R. Santos-Lima, E. M. de Gouveia Dal Pino, G. Kowal, D. Falceta-Gonçalves, A. Lazarian, M. Nakwacki, Magnetic field amplification and evolution in turbulent collisionless magnetohydrodynamics: An application to the intracluster medium. *ApJ* **781**, 84 (2014).

24. C. L. Sarazin, The energy spectrum of primary cosmic-ray electrons in clusters of galaxies and inverse compton emission. *ApJ* **520**, 529–547 (1999).

25. F. Miniati, T. W. Jones, H. Kang, D. Ryu, Cosmic-ray electrons in groups and clusters of galaxies: Primary and secondary populations from a numerical cosmological simulation. *ApJ* **562**, 233–253 (2001).

26. D. Ryu, H. Kang, E. J. Hallman, T. W. Jones, Cosmological shock waves and their role in the large-scale structure of the universe. *ApJ* **593**, 599–610 (2003).

27. G. K. Miley, The structure of extended extragalactic radio sources. *ARA&A* **18**, 165–218 (1980).

28. H. Völk, F. Aharonian, D. Breitschwerdt, The nonthermal energy content and gamma-ray emission of starburst galaxies and clusters of galaxies. *Space Sci. Rev.* **75**, 279–297 (1996).

29. A. M. Bykov, Particle acceleration and nonthermal phenomena in superbubbles. *Space Sci. Rev.* **99**, 317–326 (2001).

30. H. Kang, D. Ryu, Re-acceleration of non-thermal particles at weak cosmological shock waves. *ApJ* **734**, 18 (2011).

31. T. A. Enßlin, Gopal-Krishna, Reviving fossil radio plasma in clusters of galaxies by adiabatic compression in environmental shock waves. *A&A* **366**, 26–34 (2001).

32. E. T. Lau, A. V. Kravtsov, D. Nagai, Residual gas motions in the intracluster medium and bias in hydrostatic measurements of mass profiles of clusters. *ApJ* **705**, 1129–1138 (2009).

33. F. Vazza, G. Brunetti, A. Kritsuk, R. Wagner, C. Gheller, M. Norman, Turbulent motions and shocks waves in galaxy clusters simulated with adaptive mesh refinement. *A&A* **504**, 33–43 (2009).

34. K. Nelson, E. T. Lau, D. Nagai, Hydrodynamic simulation of non-thermal pressure profiles of galaxy clusters. *ApJ* **792**, 25 (2014).

35. T. W. Shimwell, H. J. A. Röttgering, P. N. Best, W. L. Williams, T. J. Dijkema, F. de Gasperin, M. J. Hardcastle, G. H. Heald, D. N. Hoang, A. Horneffer, H. Intema, E. K. Mahony, S. Mandal, A. P. Mechev, L. Morabito, J. B. R. Oonk, D. Rafferty, E. Retana-Montenegro, J. Sabater, C. Tasse, R. J. van Weeren, M. Brüggen, G. Brunetti, K. T. Chyży, J. E. Conway, M. Haverkorn, N. Jackson, M. J. Jarvis, J. P. McKean, G. K. Miley, R. Morganti, G. J. White, M. W. Wise, I. M. van Bemmel, R. Beck, M. Brienza, A. Bonafede, G. Calistro Rivera, R. Cassano, A. O. Clarke, D. Cseh, A. Deller, A. Drabent, W. van Driel, D. Engels, H. Falcke, C. Ferrari, S. Fröhlich, M. A. Garrett, J. J. Harwood, V. Heesen, M. Hoeft, C. Horellou, F. P. Israel, A. D. Kapińska, M. Kunert-Bajraszewska, D. J. McKay, N. R. Mohan, E. Orrú, R. F. Pizzo, I. Prandoni, D. J. Schwarz, A. Shulevski, M. Sipior, D. J. B. Smith, S. S. Sridhar, M. Steinmetz, A. Stroe, E. Varenius, P. P. van der Werf, J. A. Zensus, J. T. L. Zwart, The LOFAR two-metre Sky Survey. I. Survey description and preliminary data release. *A&A* **598**, A104 (2017).

36. T. W. Shimwell, C. Tasse, M. J. Hardcastle, A. P. Mechev, W. L. Williams, P. N. Best, H. J. A. Röttgering, J. R. Callingham, T. J. Dijkema, F. de Gasperin, D. N. Hoang, B. Hugo, M. Mirmont, J. B. R. Oonk, I. Prandoni, D. Rafferty, J. Sabater, O. Smirnov, R. J. van Weeren, G. J. White, M. Atemkeng, L. Bester, E. Bonnassieux, M. Brüggen, G. Brunetti, K. T. Chyży, R. Cochrane, J. E. Conway, J. H. Croston, A. Danezi, K. Duncan, M. Haverkorn, G. H. Heald, M. Iacobelli, H. T. Intema, N. Jackson, M. Jamrozy, M. J. Jarvis, R. Lakhoo, M. Mevius, G. K. Miley, L. Morabito, R. Morganti, D. Nisbet, E. Orrú, S. Perkins, R. F. Pizzo, C. Schrijvers, D. J. B. Smith, R. Vermeulen, M. W. Wise, L. Alegre, D. J. Bacon, I. M. van Bemmel, R. J. Beswick, A. Bonafede, A. Botteon, S. Bourke, M. Brienza, G. C. Rivera, R. Cassano, A. O. Clarke, C. J.

Conselice, R. J. Dettmar, A. Drabent, C. Dumba, K. L. Emig, T. A. Enßlin, C. Ferrari, M. A. Garrett, R. T. Génova-Santos, A. Goyal, G. Gürkan, C. Hale, J. J. Harwood, V. Heesen, M. Hoeft, C. Horellou, C. Jackson, G. Kokotanekov, R. Kondapally, M. Kunert-Bajraszewska, V. Mahatma, E. K. Mahony, S. Mandal, J. P. McKean, A. Merloni, B. Mingo, A. Miskolczi, S. Mooney, B. Nikiel-Wroczyński, S. P. O'Sullivan, J. Quinn, W. Reich, C. Roskowiński, A. Rowlinson, F. Savini, A. Saxena, D. J. Schwarz, A. Shulevski, S. S. Sridhar, H. R. Stacey, S. Urquhart, M. H. D. van der Wiel, E. Varenius, B. Webster, A. Wilber, The LOFAR Two-metre Sky Survey. II. First data release. *A&A* **622**, A1 (2019).

37. R. J. van Weeren, W. L. Williams, M. J. Hardcastle, T. W. Shimwell, D. A. Rafferty, J. Sabater, G. Heald, S. S. Sridhar, T. J. Dijkema, G. Brunetti, M. Brüggen, F. Andrade-Santos, G. A. Ogrean, H. J. A. Röttgering, W. A. Dawson, W. R. Forman, F. de Gasperin, C. Jones, G. K. Miley, L. Rudnick, C. L. Sarazin, A. Bonafede, P. N. Best, L. Bîrzan, R. Cassano, K. T. Chyży, J. H. Croston, T. Ensslin, C. Ferrari, M. Hoeft, C. Horellou, M. J. Jarvis, R. P. Kraft, M. Mevius, H. T. Intema, S. S. Murray, E. Orrú, R. Pizzo, A. Simionescu, A. Stroe, S. van der Tol, G. J. White, LOFAR facet calibration. *ApJS* **223**, 2 (2016).

38. W. L. Williams, R. J. van Weeren, H. J. A. Röttgering, P. N. Best, T. J. Dijkema, F. de Gasperin, M. J. Hardcastle, G. Heald, I. Prandoni, J. Sabater, T. W. Shimwell, C. Tasse, I. M. van Bemmel, M. Brüggen, G. Brunetti, J. E. Conway, T. Enßlin, D. Engels, H. Falcke, C. Ferrari, M. Haverkorn, N. Jackson, M. J. Jarvis, A. D. Kapińska, E. K. Mahony, G. K. Miley, L. K. Morabito, R. Morganti, E. Orrù, E. Retana-Montenegro, S. S. Sridhar, M. C. Toribio, G. J. White, M. W. Wise, J. T. L. Zwart, LOFAR 150-MHz observations of the Boötes field: Catalogue and source counts. *MNRAS* **460**, 2385–2412 (2016).

39. F. de Gasperin, T. J. Dijkema, A. Drabent, M. Mevius, D. Rafferty, R. J. van Weeren, M. Brüggen, J. R. Callingham, K. L. Emig, G. Heald, H. T. Intema, L. K. Morabito, A. R. Offringa, J. Oonk, E. Orrù, H. J. Röttgering, J. Sabater, T. W. Shimwell, A. Shulevski, W. L. Williams, Systematic effects in LOFAR data: A unified calibration strategy. *A&A* **622**, A5 (2019).

40. C. Tasse, T. W. Shimwell, M. J. Hardcastle, S. P. O’Sullivan, R. J. van Weeren, P. N. Best, L. Bester, B. Hugo, O. M. Smirnov, J. Sabater, G. Calistro-Rivera, F. de Gasperin, L. Morabito, H.

J. Röttgering, W. L. Williams, M. Bonato, M. Bondi, A. Botteon, M. Brüggen, G. Brunetti, K. T. Chyży, M. A. Garrett, G. Gürkan, M. J. Jarvis, R. Kondapally, S. Mandal, I. Prandoni, A. Repetti, E. Retana-Montenegro, D. J. Schwarz, A. Shulevski, Y. Wiaux, The LOFAR two-meter Sky Survey: Deep fields data release 1. I. direction-dependent calibration and imaging. *A&A* **648**, A1 (2021).

41. C. Tasse, Nonlinear Kalman filters for calibration in radio interferometry. *A&A* **566**, A127 (2014).

42. O. M. Smirnov, C. Tasse, Radio interferometric gain calibration as a complex optimization problem. *MNRAS* **449**, 2668–2684 (2015).

43. C. Tasse, B. Hugo, M. Mirmont, O. M. Smirnov, M. Atemkeng, L. Bester, M. J. Hardcastle, R. Lakhoo, S. Perkins, T. W. Shimwell, Faceting for direction-dependent spectral deconvolution. *A&A* **611**, A87 (2018).

44. S. van der Tol, B. D. Jeffs, A.-J. van der Veen, Self-calibration for the LOFAR radio astronomical array. *IEEE Trans. Signal Process.* **55**, 4497–4510 (2007).

45. F. de Gasperin, G. Brunetti, M. Brüggen, R. J. van Weeren, W. L. Williams, A. Botteon, V. Cuciti, T. J. Dijkema, H. Edler, M. Iacobelli, H. Kang, A. R. Offringa, E. Orrù, R. F. Pizzo, D. Rafferty, H. J. Röttgering, T. W. Shimwell, Reaching thermal noise at ultra-low radio frequencies: Toothbrush radio relic downstream of the shock front. *A&A* **642**, A85 (2020).

46. F. de Gasperin, T. J. W. Lazio, M. Knapp, Radio observations of HD80606 near planetary periastron. II. LOFAR low band antenna observations at 30-78 MHz. *A&A* **644**, A157 (2020).

47. W. L. Williams, F. de Gasperin, M. J. H. Hardcastle, R. J. van Weeren, C. Tasse, T. W. Shimwell, P. N. Best, M. Bonato, M. Bondi, M. Brüggen, H. J. A. Röttgering, D. J. B. Smith, The LOFAR LBA Sky Survey: Deep fields. I. The boötes field. *A&A* **655**, A40 (2021).

48. R. J. van Weeren, T. W. Shimwell, A. Botteon, G. Brunetti, M. Brüggen, J. M. Boxelaar, R. Cassano, G. Di Gennaro, F. Andrade-Santos, E. Bonnassieux, A. Bonafede, V. Cuciti, D. Dallacasa, F. de Gasperin, F. Gastaldello, M. J. Hardcastle, M. Hoeft, R. P. Kraft, S. Mandal, M.

Rossetti, H. J. A. Röttgering, C. Tasse, A. G. Wilber, LOFAR observations of galaxy clusters in HETDEX. Extraction and self-calibration of individual LOFAR targets. *A&A* **651**, A115 (2021).

49. A. R. Offringa, B. McKinley, N. Hurley-Walker, F. H. Briggs, R. B. Wayth, D. L. Kaplan, M. E. Bell, L. Feng, A. R. Neben, J. D. Hughes, J. Rhee, T. Murphy, N. D. R. Bhat, G. Bernardi, J. D. Bowman, R. J. Cappallo, B. E. Corey, A. A. Deshpande, D. Emrich, A. Ewall-Wice, B. M. Gaensler, R. Goeke, L. J. Greenhill, B. J. Hazelton, L. Hindson, M. Johnston-Hollitt, D. C. Jacobs, J. C. Kasper, E. Kratzenberg, E. Lenc, C. J. Lonsdale, M. J. Lynch, S. R. McWhirter, D. A. Mitchell, M. F. Morales, E. Morgan, N. Kudryavtseva, D. Oberoi, S. M. Ord, B. Pindor, P. Procopio, T. Prabu, J. Riding, D. A. Roshi, N. U. Shankar, K. S. Srivani, R. Subrahmanyan, S. J. Tingay, M. Waterson, R. L. Webster, A. R. Whitney, A. Williams, C. L. Williams, WSCLEAN: An implementation of a fast, generic wide-field imager for radio astronomy. *MNRAS* **444**, 606–619 (2014).

50. A. R. Offringa, O. M. Smirnov, An optimized algorithm for multiscale wideband deconvolution of radio astronomical images. *MNRAS* **471**, 301–316 (2017).

51. D. S. Briggs, American Astronomical Society Meeting Abstracts, in *Bulletin of the American Astronomical Society* (American Astronomical Society, 1995), vol. 27, p. 1444.

52. F. de Gasperin, W. L. Williams, P. N. Best, M. Brüggen, G. Brunetti, V. Cuciti, T. J. Dijkema, M. J. Hardcastle, M. J. Norden, A. R. Offringa, T. W. Shimwell, R. J. van Weeren, D. Bomans, A. Bonafede, A. Botteon, J. R. Callingham, R. Cassano, K. T. Chyży, K. L. Emig, H. Edler, M. Haverkorn, G. Heald, V. Heesen, M. Iacobelli, H. T. Intema, M. Kadler, K. Małek, M. Mevius, G. K. Miley, B. Mingo, L. Morabito, J. Sabater, R. Morganti, E. Orrù, R. F. Pizzo, I. Prandoni, A. Shulevski, C. Tasse, M. Vaccari, P. Zarka, H. J. Röttgering, The LOFAR LBA Sky Survey. I. Survey description and preliminary data release. *A&A* **648**, A104 (2021).

53. S. Ettori, V. Ghirardini, D. Eckert, E. Pointecouteau, F. Gastaldello, M. Sereno, M. Gaspari, S. Ghizzardi, M. Roncarelli, M. Rossetti, Hydrostatic mass profiles in X-COP galaxy clusters. *A&A* **621**, A39 (2019).

54. M. van Haarlem, M. W. Wise, A. W. Gunst, G. Heald, J. P. McKean, J. W. T. Hessels, A. G. de Bruyn, R. Nijboer, J. Swinbank, R. Fallows, M. Brentjens, A. Nelles, R. Beck, H. Falcke, R. Fender, J. Hörandel, L. V. E. Koopmans, G. Mann, G. Miley, H. Röttgering, B. W. Stappers, R. A. M. J. Wijers, S. Zaroubi, M. van den Akker, A. Alexov, J. Anderson, K. Anderson, A. van Ardenne, M. Arts, A. Asgekar, I. M. Avruch, F. Batejat, L. Bähren, M. E. Bell, M. R. Bell, I. van Bemmel, P. Bennema, M. J. Bentum, G. Bernardi, P. N. Best, L. Bîrzan, A. Bonafede, A.-J. Boonstra, R. Braun, J. Bregman, F. Breitling, R. H. van de Brink, J. Broderick, P. C. Broekema, W. N. Brouw, M. Brüggen, H. R. Butcher, W. van Cappellen, B. Ciardi, T. Coenen, J. Conway, A. Coolen, A. Corstanje, S. Damstra, O. Davies, A. T. Deller, R.-J. Dettmar, G. van Diepen, K. Dijkstra, P. Donker, A. Doorduin, J. Dromer, M. Drost, A. van Duin, J. Eislöffel, J. van Enst, C. Ferrari, W. Frieswijk, H. Gankema, M. A. Garrett, F. de Gasperin, M. Gerbers, E. de Geus, J.-M. Grießmeier, T. Grit, P. Gruppen, J. P. Hamaker, T. Hassall, M. Hoeft, H. A. Holties, A. Horneffer, A. van der Horst, A. van Houwelingen, A. Huijgen, M. Iacobelli, H. Intema, N. Jackson, V. Jelić, A. de Jong, E. Juette, D. Kant, A. Karastergiou, A. Koers, H. Kollen, V. I. Kondratiev, E. Kooistra, Y. Koopman, A. Koster, M. Kuniyoshi, M. Kramer, G. Kuper, P. Lambropoulos, C. Law, J. van Leeuwen, J. Lemaitre, M. Loose, P. Maat, G. Macario, S. Markoff, J. Masters, R. McFadden, D. McKay-Bukowski, H. Meijering, H. Meulman, M. Mevius, E. Middelberg, R. Millenaar, J. C. A. Miller-Jones, R. n. Mohan, J. D. Mol, J. Morawietz, R. Morganti, D. D. Mulcahy, E. Mulder, H. Munk, L. Nieuwenhuis, R. van Nieuwpoort, J. E. Noordam, M. Norden, A. Noutsos, A. R. Offringa, H. Olofsson, A. Omar, E. Orrù, R. Overeem, H. Paas, M. Pandey-Pommier, V. N. Pandey, R. Pizzo, A. Polatidis, D. Rafferty, S. Rawlings, W. Reich, J.-P. de Reijer, J. Reitsma, G. Renting, P. Riemers, E. Rol, J. W. Romein, J. Roosjen, M. Ruiter, A. Scaife, K. van der Schaaf, B. Scheers, P. Schellart, A. Schoenmakers, G. Schoonderbeek, M. Serylak, A. Shulevski, J. Sluman, O. Smirnov, C. Sobey, H. Spreeuw, M. Steinmetz, C. G. M. Sterks, H.-J. Stiepel, K. Stuurwold, M. Tagger, Y. Tang, C. Tasse, I. Thomas, S. Thoudam, M. C. Toribio, B. van der Tol, O. Usov, M. van Veelen, A.-J. van der Veen, S. ter Veen, J. P. W. Verbiest, R. Vermeulen, N. Vermaas, C. Vocks, C. Vogt, M. de Vos, E. van der Wal, R. J. van Weeren, H. Weggemans, P. Weltevrede, S. White, S. J. Wijnholds, T. Wilhelmsson, O. Wucknitz, S. Yatawatta, P. Zarka, J. Zensus, J. van Zwieten, LOFAR: The LOw-frequency ARray. *A&A* **556**, A2 (2013).

55. T. P. Robitaille, E. Bressert, *APLpy: Astronomical Plotting Library in Python* (Astrophysics Source Code Library, 2012).

56. G. Brunetti, *The Role of VLBI in Astrophysics, Astrometry and Geodesy*, F. Mantovani, A. Kus, Eds. (Springer, 2004), p. 29.

57. G. Brunetti, G. Setti, A. Comastri, Inverse Compton x-rays from strong FRII radio-galaxies. *A&A* **325**, 898–910 (1997).

58. A. Botteon, G. Brunetti, D. Ryu, S. Roh, Shock acceleration efficiency in radio relics. *A&A* **634**, A64 (2020).

59. F. Miniati, A. Beresnyak, Self-similar energetics in large clusters of galaxies. *Nature* **523**, 59–62 (2015).
